# Threat-related adverse childhood experiences and loneliness among middle-aged and older adults in China: indirect associations involving social activity participation breadth and functional limitations

**DOI:** 10.3389/fpubh.2026.1884393

**Published:** 2026-10-08

**Authors:** Tingke Xu, Zishuo Huang, Jiayi Liu, Qingyang Lu, Kailin Wang, Tingting Zhu, Xing Liu, Jianhui Zheng, Qiuhua Zhang, Jiajia Liu

**Affiliations:** 1 The First Affiliated Hospital of Wenzhou Medical University, Wenzhou, Zhejiang, China; 2 Department of Health Information, Sir Run Run Shaw Hospital, Zhejiang University School of Medicine, Hangzhou, Zhejiang, China; 3 School of Medical Humanities and Management, Wenzhou Medical University, Wenzhou, Zhejiang, China; 4 Business School, Renmin University of China, Beijing, China

**Keywords:** functional limitations, loneliness, middle-aged and older adults, social activity participation breadth, threat-related adverse childhood experiences

## Abstract

**Background:**

Adverse childhood experiences (ACEs) are heterogeneous, and threat-related adversities involving actual or threatened harm may be particularly relevant to later social and emotional functioning. However, evidence regarding the association between threat-related ACEs and loneliness in later life, and the potential roles of social activity participation breadth and functional limitations, remains limited among middle-aged and older Chinese adults. This study examined these associations using data from the China Health and Retirement Longitudinal Study.

**Methods:**

Data were drawn from the 2014 and 2020 China Health and Retirement Longitudinal Study (CHARLS). Threat-related ACEs—physical abuse, household substance abuse, domestic violence, unsafe neighborhood, and bullying—were retrospectively reported in 2014 and summed into a score ranging from 0 to 5. Social activity participation breadth, basic and instrumental activities of daily living (BADL/IADL) limitations, and loneliness were assessed concurrently in 2020. Although data from two CHARLS time points were linked, the specified indirect pathway was not longitudinal. Regression-based serial indirect-association models were estimated in Stata using product-of-coefficients calculations. Standard errors and 95% confidence intervals for the specific and total indirect associations were obtained from 10,000 nonparametric bootstrap replications. All estimates were interpreted as associational statistical quantities rather than causal effects.

**Results:**

Higher threat-related ACE scores were associated with higher loneliness scores. In both the IADL- and BADL-based serial indirect-association models, the largest indirect association was observed through functional limitations. The indirect associations through social activity participation breadth alone and through the specified sequential pathway were statistically distinguishable from zero but small in magnitude. The negative coefficients reflect the algebraic decomposition of fitted associations and do not indicate that greater social activity participation breadth reduces loneliness or that threat-related ACEs have a protective effect.

**Conclusion:**

Threat-related ACE scores were associated with loneliness among middle-aged and older Chinese adults. The statistical decompositions identified small indirect associations involving social activity participation breadth and functional limitations, with the largest specific indirect estimate involving functional limitations. These findings apply specifically to the five-item threat-related ACE construct used in this study and should not be generalized to deprivation-related experiences or the full range of ACEs. They do not demonstrate mediation as a temporal or causal process because the proposed intermediate variables and loneliness were measured concurrently.

## 1 Introduction

Loneliness, defined as a subjective feeling that individuals’ current social connections fall short of their desired levels, is a health issue that has often been misunderstood, neglected, or under-explored (1). This emotional state has been characterized as a signal of a unmet basic human need, akin to thirst and hunger. Nonetheless, an expanding body of evidence has demonstrated that loneliness is closely associated with adverse health outcomes (2), including substance abuse, suicidal ideation, anxiety, depression, and diminished subjective well-being (3). Moreover, a meta-analysis involving 70 studies indicated that individuals experiencing loneliness exhibited a 26% elevated risk of mortality (4).

As the global demographic structure undergoes transformative changes and the aging population intensifies, the prevalence of loneliness among middle-aged and older adults is expected to rise (5). The World Health Organization (WHO) emphasized that social isolation and loneliness were prevalent issues among middle-aged and older populations (6, 7). China boasts the world’s largest aging population, standing a chance of constituting roughly one-third of the total population by 2050 (8). Research indicated that approximately 23.4 percent of middle-aged and older adults in China experience feelings of loneliness (6). Consequently, loneliness among this demographic in China has been elevated to the status of a significant public health concern.

Adverse childhood experiences encompass heterogeneous forms of early adversity rather than a single, uniform exposure. Dimensional models distinguish threat-related experiences, which involve actual or threatened harm to physical integrity, from deprivation-related experiences, which involve the absence of expected cognitive, emotional, or social inputs (9, 10). This distinction is relevant because different adversity dimensions may show different developmental, emotional, and behavioral correlates. The present study therefore focuses specifically on threat-related adverse childhood experiences (threat-related ACEs), rather than treating the available items as a comprehensive measure of all childhood adversity.

Threat-related ACEs may be particularly relevant to later-life social and emotional functioning because experiences involving violence, intimidation, or persistent environmental danger have been associated with heightened threat sensitivity, difficulties with trust and emotion regulation, and challenges in forming or maintaining social relationships (9, 11–13). Childhood adversity has also been associated with functional limitations in middle-aged and older populations (14, 15). These findings provide a rationale for examining social activity participation breadth and functional limitations as potential intermediate factors in the association between threat-related ACEs and loneliness. Life-course theory further suggests that early adversity may be linked to later outcomes through the accumulation of social and health disadvantages over time (16).

Based on life-course theory and previous evidence linking social participation with health-related resources and functional maintenance, we examined an analytical sequence in which social activity participation breadth preceded functional limitations (17). This ordering was theory-informed rather than an established temporal sequence, and the reverse ordering is also plausible. We examined three hypotheses: (H1) higher threat-related ACE scores would be associated with greater loneliness; (H2) this association would include indirect statistical components through social activity participation breadth and BADL/IADL limitations separately; and (H3) an additional indirect component would be observed through social activity participation breadth followed by functional limitations.

## 2 Methods

### 2.1 Data source

The dataset utilized in this study was derived from the China Health and Retirement Longitudinal Study (CHARLS) conducted in 2014 and 2020. These surveys were specifically designed to target middle-aged and older adults in China, with its sampling frame encompassing households where at least one member is 45 years of age or older. To ensure sample representative, the survey followed strict randomization procedures and used a multi-stage sampling method. When sampling county and rural administrative units, a Probability-Proportional-to-Size (PPS) sampling method was adopted (14).

This study linked data from the 2014 CHARLS Life History Survey, from which threat-related ACEs were derived, with data on the remaining study variables collected in the 2020 CHARLS wave. The 2020 wave initially included 19,395 respondents. We excluded participants aged younger than 45 years (*n* = 209), those who could not be linked to the 2014 Life History Survey (*n* = 2,482), and those with missing information on loneliness (*n* = 976), social activity participation breadth (*n* = 3), BADL or IADL limitations (*n* = 2), or the selected covariates (*n* = 3,871). Because the criteria were applied sequentially, each participant was counted only once. In total, 7,543 participants were excluded, leaving 11,852 participants in the complete-case analytic sample. Figure 1 presents the participant-selection process. Although two CHARLS time points were linked, social activity participation breadth, functional limitations, and loneliness were all assessed concurrently in 2020; therefore, the specified indirect pathway was not longitudinal.

**Figure 1:**
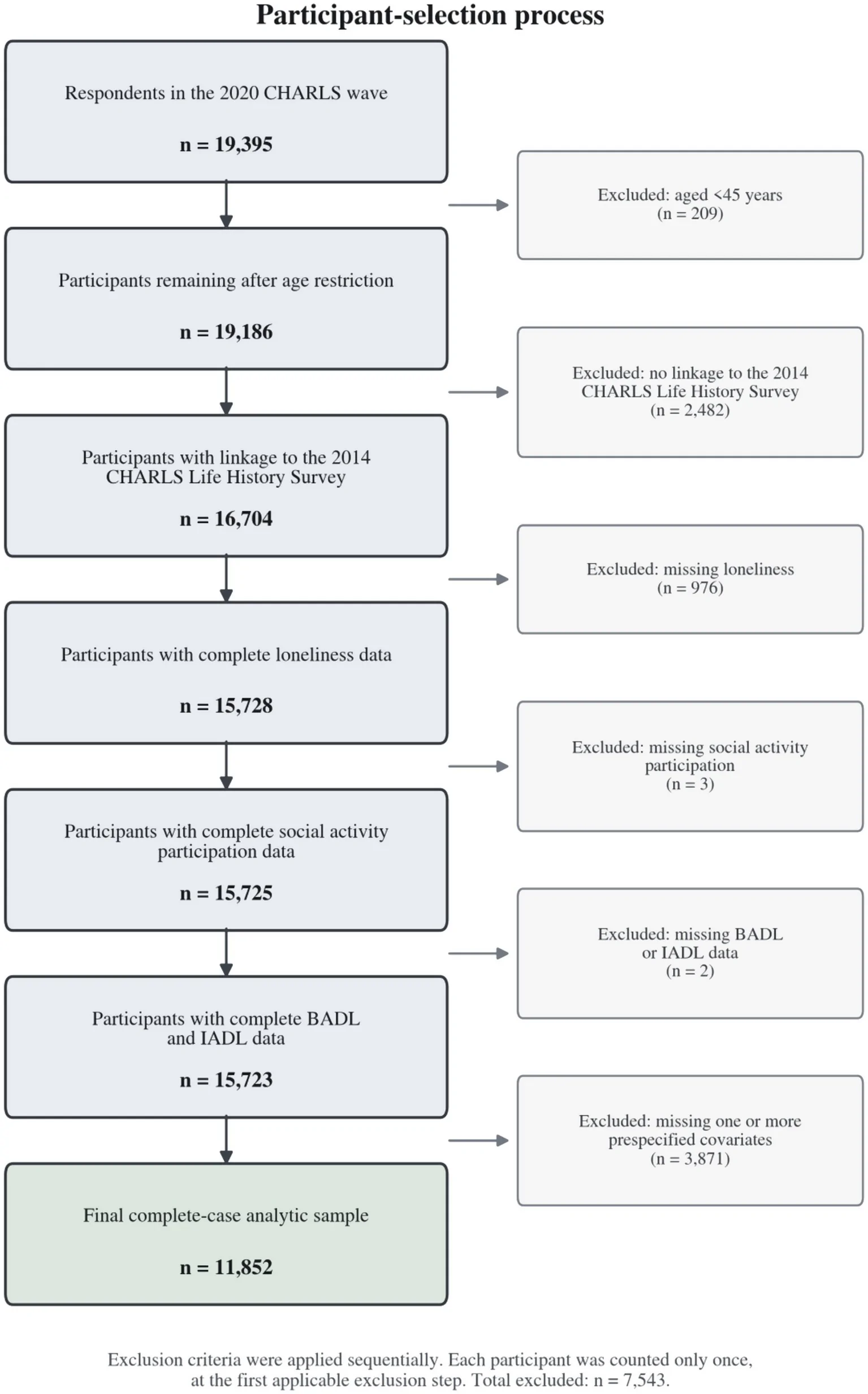
Participant-selection process. Exclusion criteria were applied sequentially. Each participant was counted only once, at the first applicable exclusion step. Total excluded: n = 7,543.

### 2.2 Measurement

#### 2.2.1 Threat-related adverse childhood experiences

Threat-related ACEs were retrospectively reported in the 2014 CHARLS life-history survey. Following the dimensional threat-versus-deprivation framework, threat refers to experiences involving actual or threatened harm, whereas deprivation refers to the absence of expected environmental inputs and caregiving (9). Following previous CHARLS-based studies, threat-related ACEs were operationalized using five childhood experiences: physical abuse, household substance abuse, domestic violence, living in an unsafe neighborhood, and bullying (15, 18). Each item was coded as 0 (no) or 1 (yes), and the items were summed to produce a cumulative threat-related ACE score ranging from 0 to 5, with higher scores indicating exposure to a greater number of threat-related adversity categories. This operationalization captures a specific threat-related subset and is not a comprehensive ACE inventory. It does not represent deprivation-related adversity or other ACE domains, such as neglect, parental separation, or household mental illness.

#### 2.2.2 Loneliness

Loneliness in Wave 5 was assessed using the single CES-D item, “I felt lonely,” which asked respondents how often they had experienced loneliness during the preceding week. Responses were coded as 0 (rarely or none of the time), 1 (some or a little of the time), 2 (occasionally or a moderate amount of the time), and 3 (most or all of the time), with higher scores indicating more frequent feelings of loneliness. This item has been used as a brief indicator of loneliness in previous CHARLS-based studies and is suitable for minimizing respondent burden in large population surveys (19). Validation studies have found that direct single-item loneliness measures demonstrate moderate convergence with established multi-item loneliness scales and expected associations with depression, anxiety, life satisfaction, and social-relationship characteristics (20–22). Nevertheless, the single item should be interpreted as a brief measure of the frequency of perceived loneliness rather than a comprehensive assessment of its social and emotional dimensions.

#### 2.2.3 Social activity participation breadth

Social activity participation breadth in Wave 5 was operationalized as the breadth of social activity participation during the preceding month. Respondents were asked whether they had participated in any of eight activity categories: socializing with friends; playing cards, mahjong, or chess, or attending a community club; providing help to relatives, friends, or neighbors; dancing, exercising, or practicing qigong; participating in community organizations; undertaking voluntary or charitable activities; attending educational or training courses; and other social activities. Each category was coded as 0 (no) or 1 (yes) and summed to generate a social activity participation breadth score ranging from 0 to 8. Higher scores indicate participation in a wider variety of social activities, whereas a score of 0 indicates no participation in the listed activities during the preceding month. Similar activity-count measures have been used in previous CHARLS-based studies, and prior research has distinguished the diversity of activity types from participation frequency when examining the health relevance of social participation among middle-aged and older adults (23–25). The included categories cover several common informal, recreational, mutual-help, physical, organizational, voluntary, and educational forms of participation in this population. Accordingly, the score should be interpreted as an indicator of participation breadth or diversity rather than frequency, duration, intensity, relationship quality, social role, or subjective meaning.

#### 2.2.4 Functional limitations

In Wave 5, functional limitations were assessed separately using basic activities of daily living (BADL) and instrumental activities of daily living (IADL). BADL comprised six basic self-care activities: dressing, bathing or showering, eating, getting into or out of bed, using the toilet, and controlling urination and defecation (26). IADL comprised five activities necessary for independent living: shopping for groceries, preparing hot meals, performing household chores, managing money, and taking medications (27). For each activity, respondents reported whether they had no difficulty, had difficulty but could still perform the activity independently, had difficulty and required assistance, or were unable to perform the activity. Responses were scored from 1 to 4, respectively, according to increasing levels of difficulty, and item scores were summed separately (28). Total scores ranged from 6 to 24 for BADL and from 5 to 20 for IADL, with higher scores indicating greater functional limitations. Because BADL and IADL represent related but distinct dimensions of daily functioning, they were analyzed separately and are consistently described as measures of functional limitations throughout the manuscript.

#### 2.2.5 Covariates

Covariates were selected on the basis of the life-course framework and previous evidence regarding factors associated with childhood adversity, social activity participation breadth, functional limitations, or loneliness among middle-aged and older adults (29–32). The adjusted models included age (45–59/≥60 years), gender (female/male), residence (rural/urban), education (illiterate/literate), marital status (without/with a spouse), smoking (no/yes), drinking (no/yes), and exercise (no/yes), thereby accounting for selected demographic, socioeconomic, family, and health-behavior characteristics. The same covariate set was included in all regression equations to maintain consistency between the IADL and BADL models. Covariate adjustment was used to estimate associations conditional on these measured characteristics; it should not be interpreted as eliminating confounding or establishing causal effects, because residual and unmeasured confounding remains possible.

### 2.3 Statistical analysis

All analyses were conducted in Stata (StataCorp LLC, College Station, TX, USA). Continuous variables are presented as means and standard deviations, and categorical variables as frequencies and percentages. Differences in loneliness across participant characteristics were examined using independent-samples t tests. Spearman correlation coefficients were calculated for threat-related ACEs, social activity participation breadth, BADL/IADL limitations, and loneliness. Regression-based serial indirect-association models were then estimated separately for IADL and BADL using a product-of-coefficients approach. Social activity participation breadth was entered as the first intermediate variable and functional limitations as the second. Each model estimated the total association, the association conditional on both intermediate variables, the two single-intermediate-variable indirect associations, and the sequential indirect association. The total indirect association was calculated as the sum of the three indirect estimates. Standard errors and 95% confidence intervals for indirect associations were obtained from 10,000 nonparametric bootstrap replications. All models adjusted for age, gender, residence, education, marital status, smoking, drinking, and exercise. Gender was included only as an adjustment covariate; gender-specific indirect associations and between-gender comparisons were not examined because they were outside the primary scope of this study. Analyses were restricted to complete cases and did not incorporate CHARLS sampling weights, clustering, or stratification. Accordingly, the estimates apply to the analytic sample rather than representing fully design-based national estimates. The terms ‘direct,’ ‘indirect,’ and ‘sequential’ are conventional statistical labels within this associational framework and do not establish temporal ordering or causal effects. All tests were two-sided, with *p* < 0.05 considered statistically significant.

## 3 Results

The analytic sample included 11,852 middle-aged and older adults, of whom 46.98% were men and 76.95% lived in rural areas. Most participants were literate (78.59%), lived with a spouse (85.39%), did not smoke (74.16%), did not drink (63.58%), and reported exercise (77.78%). Mean loneliness scores differed by gender, age group, residence, education, marital status, smoking, and drinking (all *p* < 0.001), but not by exercise status (t = 1.857, *p* = 0.063; Table 1). Mean scores were 1.30 ± 1.04 for threat-related ACEs, 5.75 ± 2.05 for IADL limitations, 6.55 ± 1.52 for BADL limitations, 0.79 ± 1.00 for social activity participation breadth, and 0.60 ± 1.01 for loneliness.

**Table 1:** Differences in loneliness according to participant characteristics.

Characteristic	*n* (%)	Loneliness, mean ± SD	t	*p*
Gender			10.241	<0.001
Female	6,284 (53.02)	0.69 ± 1.06		
Male	5,568 (46.98)	0.50 ± 0.93		
Age group			−7.119	<0.001
45–59 years	5,250 (44.30)	0.53 ± 0.94		
≥60 years	6,602 (55.70)	0.66 ± 1.06		
Residence			9.680	<0.001
Rural	9,120 (76.95)	0.65 ± 1.04		
Urban	2,732 (23.05)	0.44 ± 0.87		
Education			11.974	<0.001
Illiterate	2,538 (21.41)	0.81 ± 1.14		
Literate	9,314 (78.59)	0.54 ± 0.96		
Marital status			24.684	<0.001
Without a spouse	1,731 (14.61)	1.14 ± 1.25		
With a spouse	10,121 (85.39)	0.51 ± 0.93		
Smoking			3.733	<0.001
No	8,789 (74.16)	0.62 ± 1.02		
Yes	3,063 (25.84)	0.54 ± 0.97		
Drinking			8.353	<0.001
No	7,535 (63.58)	0.66 ± 1.04		
Yes	4,317 (36.42)	0.50 ± 0.93		
Exercise			1.857	0.063
No	2,634 (22.22)	0.63 ± 1.04		
Yes	9,218 (77.78)	0.59 ± 1.00		

Table 2 presents the descriptive statistics and Spearman correlations for the focal variables. Threat-related ACE scores showed weak positive correlations with IADL limitations (r_s_ = 0.020, *p* < 0.05), BADL limitations (r_s_ = 0.027, *p* < 0.01), and loneliness (r_s_ = 0.075, *p* < 0.01), and a weak negative correlation with social activity participation breadth (r_s_ = −0.087, *p* < 0.01). Greater participation breadth was weakly associated with fewer IADL limitations (r_s_ = −0.106), fewer BADL limitations (r_s_ = −0.082), and lower loneliness (r_s_ = −0.050; all *p* < 0.01). IADL and BADL limitations were strongly correlated (r_s_ = 0.664, *p* < 0.01), and both were positively correlated with loneliness (r_s_ = 0.208 and 0.206, respectively; both *p* < 0.01). These correlations describe unadjusted bivariate relationships and should not be interpreted as evidence of direction or causation.

**Table 2:** Spearman correlations among threat-related ACEs, functional limitations, social activity participation breadth, and loneliness.

Variable	Mean ± SD	Threat-related ACEs	IADL	BADL	Social activity participation breadth	Loneliness
Threat-related ACEs	1.30 ± 1.04	1.000				
IADL limitations	5.75 ± 2.05	0.020*	1.000			
BADL limitations	6.55 ± 1.52	0.027**	0.664**	1.000		
Social activity participation breadth	0.79 ± 1.00	−0.087**	−0.106**	−0.082**	1.000	
Loneliness	0.60 ± 1.01	0.075**	0.208**	0.206**	−0.050**	1.000

Threat-related ACEs = the five-item threat-related adverse childhood experience score; BADL = basic activities of daily living; IADL = instrumental activities of daily living. Higher BADL/IADL scores indicate greater functional limitations. *p < 0.05; **p < 0.01.

Table 3 presents the results of the regression-based serial indirect-association models. In the IADL model, the total association between threat-related ACEs and loneliness was 0.1021 (SE = 0.0087, 95% CI: 0.0850 to 0.1192), and the corresponding association conditional on social activity participation breadth and IADL limitations was 0.0925 (SE = 0.0086, 95% CI: 0.0757 to 0.1094). The total indirect association was 0.0095 (bootstrap SE = 0.0021, 95% CI: 0.0058 to 0.0140). The specific indirect estimates were −0.0010 through social activity participation breadth, 0.0111 through IADL limitations, and −0.0006 through social activity participation breadth followed by IADL limitations. The BADL model showed a similar pattern. The total association was 0.1021 (SE = 0.0087, 95% CI: 0.0850 to 0.1192), whereas the association conditional on social activity participation breadth and BADL limitations was 0.0926 (SE = 0.0086, 95% CI: 0.0757 to 0.1095). The total indirect association was 0.0094 (bootstrap SE = 0.0018, 95% CI: 0.0059 to 0.0130), comprising specific indirect estimates of −0.0012 through social activity participation breadth, 0.0110 through BADL limitations, and −0.0004 through social activity participation breadth followed by BADL limitations. The bootstrap confidence intervals for all reported indirect associations excluded zero. In both models, the indirect association through functional limitations was the largest in absolute magnitude, although all indirect estimates were small. The total indirect estimates of 0.0095 and 0.0094 were approximately 9.3 and 9.2% of the total association of 0.1021, respectively, while both sequential components were below 0.001. These small components, although statistically detectable in this large sample, should not be interpreted as clinically meaningful individual-level effects. The negative coefficients are algebraic products of the fitted associations within the decomposition and do not indicate a protective effect of threat-related ACEs or a causal benefit of greater social activity participation breadth. Figure 2 presents the corresponding path coefficients. These estimates do not establish temporal ordering or causal mediation.

**Table 3:** Results of the serial indirect-association models of threat-related ACEs and loneliness.

Model	Association component	Pathway	Estimate	SE	95% CI	Standardized estimate (95% CI)
IADL	Conditional direct association	Threat-related ACEs → Loneliness	0.0925	0.0086	0.0757, 0.1094	0.0952 (0.0779, 0.1126)
IADL	Specific indirect association	Threat-related ACEs → Social activity participation breadth → Loneliness	−0.0010	0.0005	−0.0020, −0.0001	−0.0010 (−0.0021, −0.0001)
IADL	Specific indirect association	Threat-related ACEs → IADL limitations → Loneliness	0.0111	0.0018	0.0077, 0.0148	0.0114 (0.0079, 0.0152)
IADL	Sequential indirect association	Threat-related ACEs → Social activity participation breadth → IADL limitations → Loneliness	−0.0006	0.0001	−0.0009, −0.0004	−0.0006 (−0.0009, −0.0004)
IADL	Total indirect association	Sum of the three indirect associations	0.0095	0.0021	0.0058, 0.0140	0.0098 (0.0060, 0.0144)
IADL	Total association	Overall threat-related ACEs–loneliness association	0.1021	0.0087	0.0850, 0.1192	0.1051 (0.0875, 0.1227)
BADL	Conditional direct association	Threat-related ACEs → Loneliness	0.0926	0.0086	0.0757, 0.1095	0.0954 (0.0779, 0.1128)
BADL	Specific indirect association	Threat-related ACEs → Social activity participation breadth → Loneliness	−0.0012	0.0005	−0.0022, −0.0003	−0.0012 (−0.0023, −0.0003)
BADL	Specific indirect association	Threat-related ACEs → BADL limitations → Loneliness	0.0110	0.0018	0.0076, 0.0145	0.0113 (0.0078, 0.0149)
BADL	Sequential indirect association	Threat-related ACEs → Social activity participation breadth → BADL limitations → Loneliness	−0.0004	0.0001	−0.0006, −0.0002	−0.0004 (−0.0006, −0.0002)
BADL	Total indirect association	Sum of the three indirect associations	0.0094	0.0018	0.0059, 0.0130	0.0097 (0.0061, 0.0134)
BADL	Total association	Overall threat-related ACEs–loneliness association	0.1021	0.0087	0.0850, 0.1192	0.1051 (0.0875, 0.1227)

All models adjusted for age, gender, residence, education, marital status, smoking, drinking, and exercise. Confidence intervals for the specific and total indirect associations were based on 10,000 nonparametric bootstrap replications. “Direct,” “indirect,” and “sequential” are conventional statistical labels and do not imply temporal ordering or causal effects. IADL = instrumental activities of daily living; BADL = basic activities of daily living; ACEs = adverse childhood experiences.

**Figure 2:**
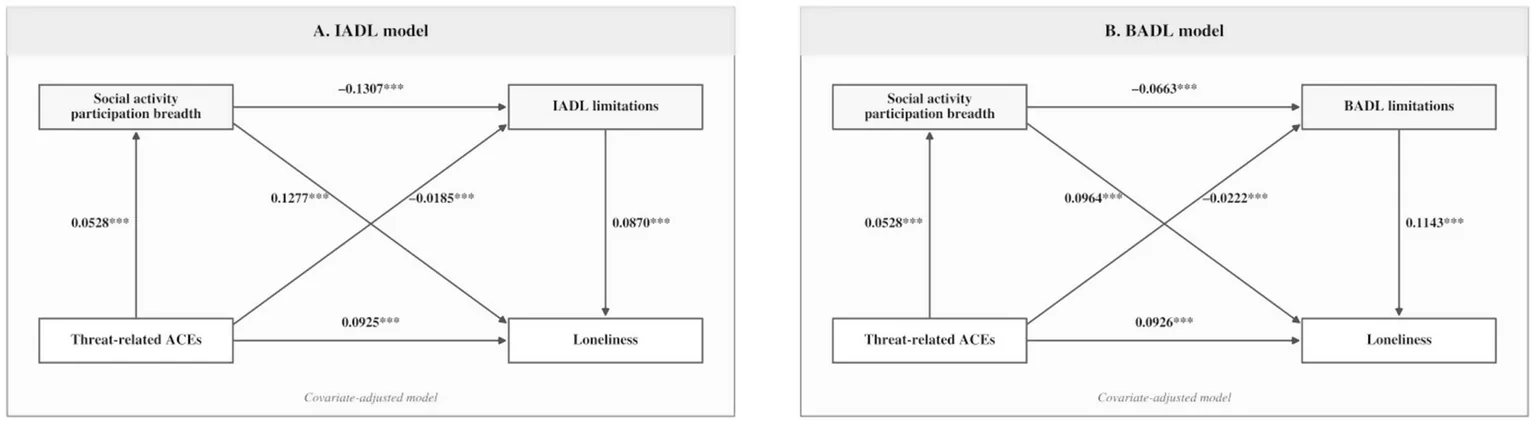
Path coefficients for the IADL and BADL serial indirect-association models. Values beside the arrows are unstandardized regression coefficients. IADL = instrumental activities of daily living; BADL = basic activities of daily living; threat-related ACEs = the five-item threat-related adverse childhood experience score. Social activity participation breadth indicates the number of activity categories reported during the preceding month. Higher BADL and IADL scores indicate greater functional limitations. Both models adjusted for age, gender, residence, education, marital status, smoking, drinking, and exercise. The ordering of variables reflects the specified analytical model and should not be interpreted as an established temporal or causal sequence. ***p < 0.001.

## 4 Discussion

To our knowledge, this is the first study to examine the association between threat-related ACEs and loneliness among middle-aged and older Chinese adults while considering social activity participation breadth and functional limitations within a statistical framework for serial indirect associations. Higher threat-related ACE scores were associated with greater loneliness. A small proportion of this association was statistically accounted for by differences in social activity participation breadth and functional limitations. The indirect association through BADL or IADL limitations was larger than those through social activity participation breadth alone or through social activity participation breadth followed by functional limitations, although all indirect estimates were small. By distinguishing threat-related ACEs from other forms of childhood adversity, this study provides a more specific understanding of how these experiences are associated with loneliness in later life. However, the findings are limited to the five threat-related experiences available in CHARLS, and the indirect associations should not be interpreted as evidence of temporal or causal mechanisms.

The observed positive association between threat-related ACEs and loneliness is consistent with previous evidence linking childhood maltreatment and violence exposure with loneliness, poorer mental health, and difficulties in social and interpersonal functioning during adulthood (33–35). Life-course and cumulative-inequality perspectives suggest that childhood experiences may remain relevant to later-life health and social relationships through interconnected psychological, social, and socioeconomic pathways that accumulate over time (36). Threat-related experiences may also be associated with difficulties in perceived safety, emotional regulation, and interpersonal trust, which could be relevant to the development and maintenance of social relationships. Among middle-aged and older Chinese adults, later-life transitions such as retirement, bereavement, family separation, and reduced access to social resources may further influence loneliness. However, these potential mechanisms and contextual factors were not directly measured in this study and should therefore be regarded as possible explanations rather than established pathways.

The indirect association through social activity participation breadth was negative and very small. In the adjusted model, higher threat-related ACE scores were associated with slightly greater social activity participation breadth, whereas greater social activity participation breadth was associated with lower loneliness. This pattern differed from the negative unadjusted correlation, indicating that the estimate was sensitive to covariate adjustment and should be interpreted cautiously. Previous studies have linked childhood adversity to later difficulties in trust, attachment, perceived social support, and social connection (37–39). Moreover, the activity-count score reflects the variety of activities rather than their frequency, quality, or emotional significance. Although the indirect estimate was small, threat-related childhood adversity and loneliness affect substantial population groups, meaning that small individual-level associations may still have potential public health relevance. However, this implication requires confirmation, and statistical significance in a large sample should not be interpreted as evidence of clinical importance or a causal mechanism.

The indirect associations through BADL and IADL limitations were larger than that through social activity participation breadth, although their absolute magnitudes remained small. This pattern is consistent with previous studies reporting associations between childhood adversity and functional limitations in later life (40, 41). Threat-related childhood experiences may remain associated with physical and psychological health across adulthood, which could be relevant to difficulties in performing daily activities. BADL limitations reflect difficulties in basic self-care, whereas IADL limitations affect more complex activities required for independent living and interaction with the broader social environment. Greater functional limitations may coincide with reduced mobility, loss of independence, increased reliance on others, and fewer opportunities to maintain social relationships, thereby contributing to greater vulnerability to loneliness. From a public health perspective, even modest associations may be relevant because functional limitations and loneliness are common concerns among aging populations and may jointly identify individuals requiring additional health and social support (42). Nevertheless, the relationship may be bidirectional: loneliness may also be associated with lower activity levels and poorer health behaviors, while both loneliness and functional limitations may reflect shared factors such as chronic disease, socioeconomic disadvantage, depression, or cognitive impairment. Because functional limitations and loneliness were assessed concurrently, the direction and temporal ordering of these associations cannot be determined.

The sequential indirect associations through social activity participation breadth followed by functional limitations were statistically distinguishable from zero but very small in absolute magnitude (IADL: −0.0006; BADL: −0.0004). Previous studies have associated social activity participation breadth with physical activity, access to information, practical assistance, and social support, which may be relevant to the maintenance of daily functioning (17, 43). This evidence provides a theoretical basis for positioning social activity participation breadth before functional limitations in the analytical model. However, the reverse ordering is also plausible because functional limitations may restrict access to social and community activities. Social activity participation breadth and functional limitations may also change reciprocally over time or reflect shared health and socioeconomic circumstances. Therefore, the negative sequential estimates should not be interpreted as indicating that threat-related ACEs have a protective association with loneliness. Instead, they represent very small components of the regression-based statistical decomposition. Longitudinal studies with repeated assessments of social activity participation breadth, functional limitations, and loneliness are needed to examine their temporal ordering and possible reciprocal relationships.

Although the indirect associations were statistically distinguishable from zero, their magnitudes were small, particularly those involving social activity participation breadth and the sequential pathway. In quantitative terms, the total indirect estimates represented approximately 9.3% in the IADL model and 9.2% in the BADL model of the corresponding total association, and the sequential components were below 0.001. Their detectability in 11,852 participants therefore does not by itself imply a meaningful individual-level or clinical effect. Given the observational design, these findings should be interpreted cautiously and do not demonstrate that increasing social activity participation breadth or reducing functional limitations would necessarily alleviate loneliness. Nevertheless, the findings may inform person-centered assessment in geriatric, primary-care, and community settings, where loneliness, barriers to social activity participation breadth, and functional limitations could be considered together to identify individual support needs. When discussing childhood adversity, healthcare professionals should adopt a trauma-informed approach that respects consent, privacy, personal choice, and psychological safety. BADL and IADL assessments may also help identify potential needs for rehabilitation, assistive devices, or social-care services. Prospective intervention studies are needed to determine whether these approaches can reduce loneliness.

## 5 Conclusion

Higher threat-related ACE scores were associated with greater loneliness among middle-aged and older Chinese adults. By distinguishing threat-related experiences from the broader and heterogeneous ACE construct, this study provides more specific evidence on the relationship between childhood adversity and loneliness in later life. Small indirect associations were observed through social activity participation breadth and functional limitations, with BADL and IADL limitations showing the largest indirect estimates. These findings extend the existing literature by integrating childhood adversity, social activity participation breadth, functional limitations, and loneliness within a single analytical framework and highlight the potential value of considering social and functional circumstances in later-life research and person-centered assessment. However, the findings are limited to the five-item threat-related ACE measure available in CHARLS, and the concurrent assessment of the intermediate variables and loneliness precludes conclusions about temporal ordering or causal mediation.

## 6 Limitation

This study has several limitations. First, social activity participation breadth, BADL/IADL limitations, and loneliness were assessed concurrently; therefore, their temporal ordering cannot be determined, and the indirect associations should not be interpreted as causal mediation. Second, reliance on self-reported data may have introduced recall bias, social desirability, measurement error, and common-source bias. The single-item loneliness measure may not capture the multidimensional nature of loneliness, the five-item threat-related ACE score did not represent the full range or characteristics of childhood adversity, and the social activity participation breadth measure captured activity types rather than their frequency, quality, or personal meaning. Third, the complete-case analysis excluded 7,543 participants without formally comparing those with complete and incomplete data, so selection bias remains possible. Because exclusions were partly due to missing values in the focal study variables, a complete comparison across all main study variables was not possible. Sampling weights, clustering, and stratification were not incorporated; consequently, the estimates should not be regarded as fully design-based national estimates. Residual confounding cannot be excluded, and gender-specific associations were not formally examined. Finally, the indirect estimates were small, and their practical importance remains uncertain. Future studies should use longitudinal designs with repeated measurements, multidimensional measures of loneliness and social activity participation breadth, more comprehensive assessments of threat-related ACEs, formal evaluation of gender differences, and analytical methods that account for the complex survey design. Such studies would help clarify temporal relationships, assess potential population heterogeneity, and determine whether the observed associations have meaningful implications for intervention and service planning.

## Data Availability

Publicly available datasets were analyzed in this study. This data can be found: All CHARLS data are publicly available and can be requested on the website (https://charls.pku.edu.cn/).
